# Anatomical Variant of the Lateral Circumflex Femoral Artery: A Cadaveric Case Report

**DOI:** 10.7759/cureus.52117

**Published:** 2024-01-11

**Authors:** Mason P Granger, Megan E Gremillion, Driskell R Greene, Tristan M Halbrook, Sanjeev S Gummandi, Adegbenro O Fakoya

**Affiliations:** 1 School of Medicine, Louisiana State University Health Sciences Center, Shreveport, USA; 2 Cellular Biology and Anatomy, Louisiana State University Health Sciences Center, Shreveport, USA

**Keywords:** cadaver, femoral artery, lateral circumflex femoral artery, deep femoral artery, anatomical variant, anatomy

## Abstract

The lateral circumflex femoral artery (LCFA), a branch of the deep femoral artery (DFA), supplies the muscular and fascial anatomy of the anterior thigh. An undocumented variation of the LCFA was discovered during a dissection of the lower extremities. The LCFA is a vital vessel that can be used in coronary artery bypass grafts (CABGs) and reconstructive and bypass surgical procedures. On the other hand, the LCFA is susceptible to iatrogenic damage during surgeries involving the hip joint and procedures such as femoral nerve blocks. Knowledge of variations in the origin and course of the LFCA, like many other anatomical structures, is an important concept that physicians and health care providers must be aware of when performing anterior thigh procedures. This case report shows an interesting duplication of the LCFA, the first originating superiorly from the common femoral artery (CFA) and the second from the deep femoral artery (DFA) inferiorly. Both LCFAs exhibited typical trifurcation into ascending, descending, and transverse branches.

## Introduction

The common femoral artery (CFA), commonly referred to as the femoral artery, serves as the principal arterial supply to the lower extremities [1]. The deep femoral artery (DFA), also known as the profunda femoris, originates from the CFA and serves as the main continuation of the CFA. Two major arteries branch from the DFA: the medial circumflex femoral artery (MCFA) and the lateral circumflex femoral artery (LCFA) [2]. Along its path, the LCFA passes deep to the rectus femoris and sartorius muscles in the lateral aspect of the thigh. It then gives way to three main arteries: the ascending, transverse, and descending branches. The ascending branch supplies the anterolateral part of the gluteal region and the greater trochanter of the femur, the transverse branch wraps around the neck of the femur and contributes to the cruciate anastomosis, and the descending branch runs deep to the rectus femoris and medial to vastus lateralis all the way up to the knee joint to join the genicular anastomosis [3]. A study reported that the LCFA also contributes to the vascular supply of the sciatic nerve in up to 25% of dissected cadavers [4].

Anatomical variations in the LCFA have been extensively documented. The most common branch point of the artery is one in which the LCFA originates from the DFA [3,5]. Less common branch points of the LCFA are from the CFA or superficial femoral artery [3,5]. There have also been documented cases in which the LCFA originates with the DFA at a common stem from the CFA [3,5]. Rarely does LCFA originate from the external iliac artery (EIA) [3,5]. Duplications of the LCFA have been documented as well [3,5]. There have been cases in which the duplicated LCFAs have both branched from the DFA, one from the DFA and one from the CFA, one from the DFA and one from its branch point, and one from the CFA and the common stem of the DFA [3,5]. Origins of the ascending and descending branches vary as well. Observed branch points for both the ascending and descending branches include the CFA, DFA, and LCFA [6].

The predominant observed branching pattern, constituting 50% of cases, involves the MCFA and LCFA branching from the DFA [7]. In 31% of the population, the MCFA originates from the common or superficial femoral artery (SFA) while the LCFA arises from the DFA [7]. Alternatively, in 15% of cases, the MCFA originates from the DFA while the LCFA originates from the CFA or SFA. To summarize, the MCFA originates from the CFA in 31%, from the DFA in 65%, and from the SFA in 3% of cases. Regarding the LCFA, its origin is from the CFA in 9.7% of cases and from the DFA in 90.3% of cases [7].

Given the increasing utilization of the LCFA in surgical procedures, particularly for grafting purposes, understanding the variations and anatomical intricacies within this vascular region has gained significant relevance. This case report specifically explores the duplicated variant of the LCFA and its ascending, descending, and transverse branches originating from both the CFA and DFA, identified during a routine cadaveric dissection, shedding light on its clinical implications.

## Case presentation

**Figure 1:**
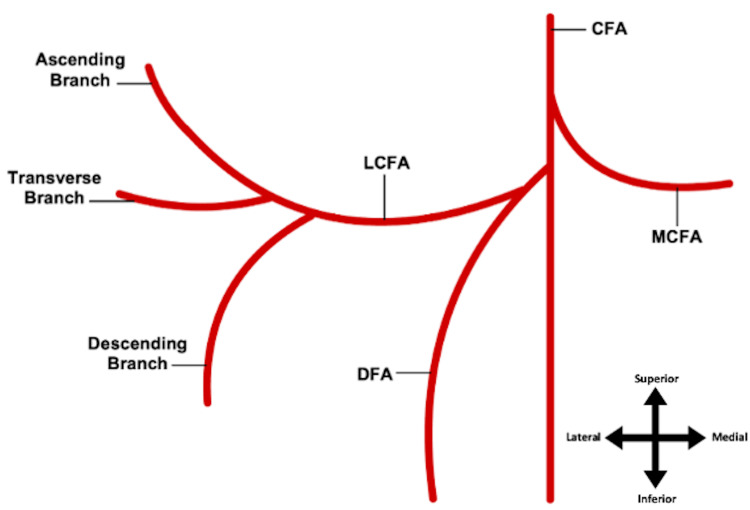
Typical anatomy of the origin of the LCFA The LCFA typically arises from the DFA with its three branches: ascending, transverse, and descending LCFA: lateral circumflex femoral artery; DFA: deep femoral artery Image Credits: Megan E. Gremillion

**Figure 2:**
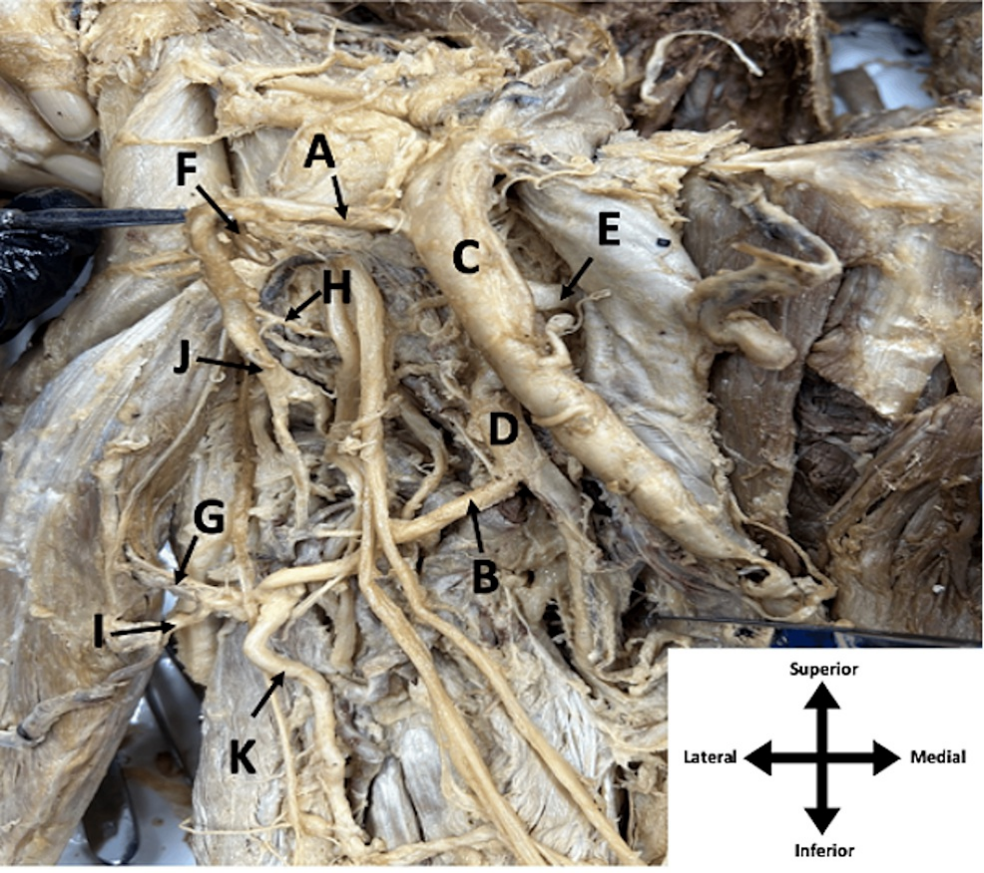
Femoral triangle of the right leg of an elderly male cadaver Duplicated LCFAs (A and B), CFA (C), DFA (D), MCFA (E), duplicated ascending branches (F and G), duplicated transverse branches (H and I), and duplicated descending branches (J and K) are shown. LCFA: lateral circumflex femoral artery; CFA: common femoral artery; DFA: deep femoral artery; MCFA: medial circumflex femoral artery

**Figure 3:**
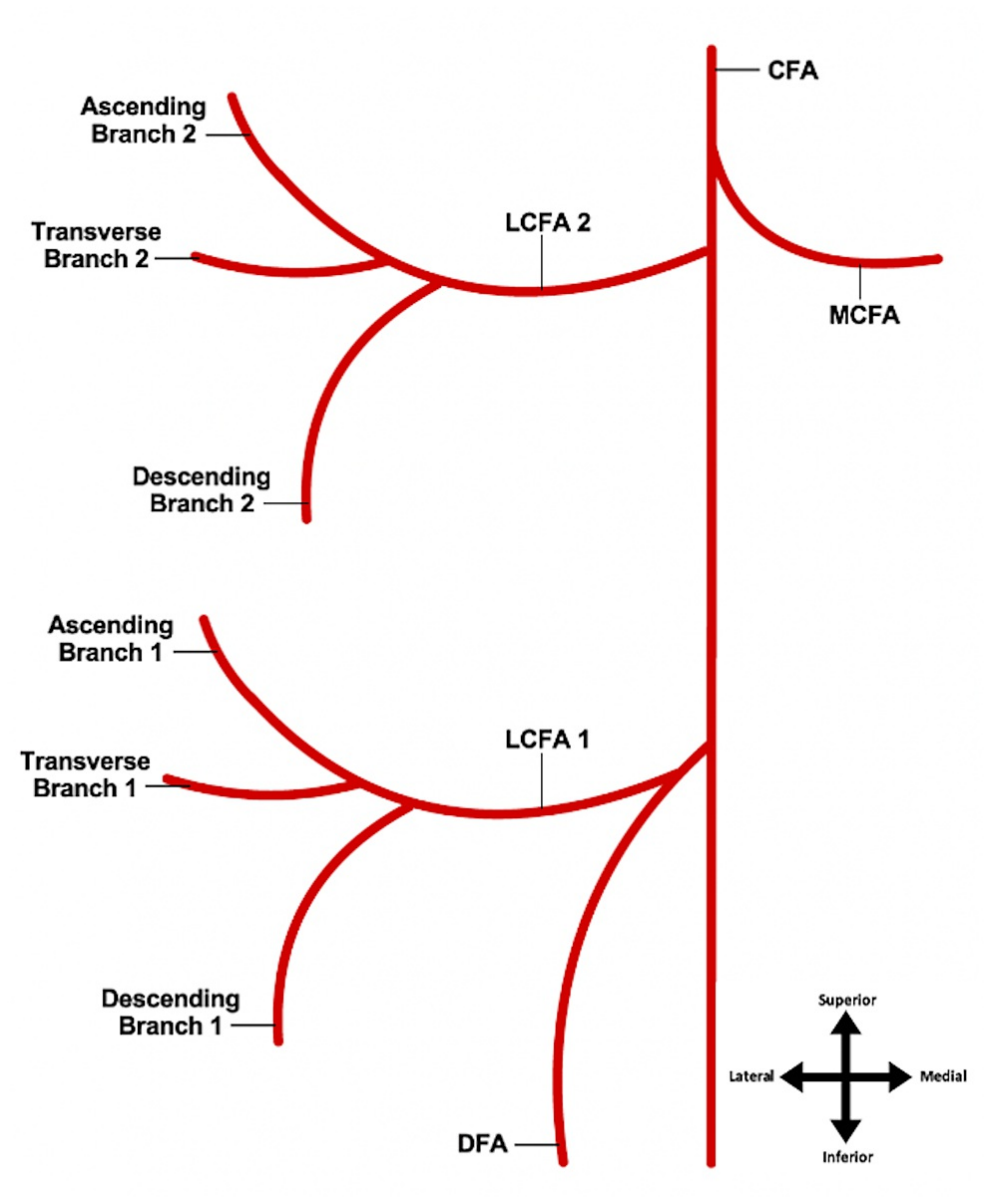
Duplication of the LCFA and its branches LCFA 1 arising from the DFA being the typical course, and LCFA 2 being the abnormal duplication emerging from the CFA LCFA: lateral circumflex femoral artery; DFA: deep femoral artery; CFA: common femoral artery Image Credits: Megan E. Gremillion

During our anatomical dissection as medical students at Louisiana State University Health Shreveport School of Medicine, we encountered distinctive LCFA variation within the right lower limb of a 75-year-old male cadaver. There were no obvious incisions or surgical scars seen. Conventionally, the LCFA stems from the DFA, dividing into ascending, transverse, and descending branches [2,3], as shown in Figure 1. Our dissection unveiled a remarkably rare occurrence: a duplication of the LCFA and all three of its branches, constituting the first variation of this type to our knowledge. Moreover, the duplicated LCFAs originated from disparate sites, as evident in Figure 2 and Figure 3: the first LCFA (A) arose superiorly from the CFA (C) and the second LCFA (B) originated inferiorly from the DFA (D). Uniquely, both sets of LCFAs exhibited the typical trifurcation into ascending, descending, and transverse branches, marking a complete duplication of the LCFA and its associated branches in this cadaver. Intriguingly, this anomaly was exclusive to the right lower limb, not the left lower limb. Additionally, as depicted in Figure 2 and Figure 3, the CFA gave rise to the MCFA (E), responsible for femoral head vascularization in conjunction with the LCFA, exhibiting a typical course in this particular specimen.

## Discussion

The literature describes inter-cadaveric and interpatient variability in the course of the LCFA, as the vessel holds great importance in terms of surgical procedures [8]. The most commonly observed variations include the LCFA originating from the DFA rather than the CFA [3,9], as well as major branches of the LCFA originating from the DFA directly [3]. However, the aberrant course of the LCFA with duplication, as described in the current report, is novel. Tomaszewski et al. performed a meta-analysis describing and documenting numerous variations of the LCFA to propose a new classification system for the origins of the LCFA. The LCFA was observed branching from the CFA, DFA, and superficial femoral artery. The ascending and descending branches of the LCFA were also documented as having various branch points from the LCFA, DFA, or CFA [6]. In contrast, Ma et al. dissected 58 adult cadavers with 115 intact sides to describe branch pattern variations of the LCFA. Subtypes were lettered A-G and included specimens with duplications of the LCFA [5]. Unlike previously documented variations, the LCFA detailed in this case report and its ascending, descending, and transverse branches were duplicated and had different origins, one from the CFA superiorly and the other from the DFA inferiorly, as shown in Figure 2 and Figure 3. In this report, the replication of the LCFA resembles Ma et al.'s Type-E yet differs in the branching location. While Ma and colleagues observed an inferior branching pattern from the CFA, here, the LCFA branches out superiorly from the same source [5]. In addition, the study by Ma et al. only considered the origin of the LCFA with no reference to the offshoot branches, as done by Tomaszewski and colleagues [5,6].

The LCFA, specifically its descending branch, provides an efficacious alternative arterial graft to the saphenous vein for coronary artery bypass graft (CABG) procedures [8]. Additionally, the descending branch of the LCFA has been used successfully in reconstructive surgeries via anterolateral thigh flaps [10]. Furthermore, the clinical relevance of the LCFA is broadened immensely due to its origin, as damage to either the CFA or DFA has implications for the viability of the LCFA. The CFA provides a viable alternative for vascular hemodialysis access [11], and the DFA is advantageous for its use as a flap in breast reconstruction surgeries [12]. Thus, the LCFA and its course in the human body are crucial for medical professionals in preventing surgical complications in patients undergoing CABG, reconstructive surgeries, hemodialysis, and various additional procedures [5].

This uncommon anatomical course further exemplifies the need for angiographic studies prior to surgical procedures involving the LCFA, the structures it supplies, or the vessel it originates from, as operations involving the LCFA without awareness regarding interpatient variations in the vessel could result in disruption of blood supply to soft tissues of the hip joint, knee joint [3], tensor fasciae latae muscle, quadriceps femoris muscle, and skin of the anterolateral thigh [1]. Notably, the LCFA possesses an anastomotic relationship with the MCFA to supply the femoral head [13]. As such, injuries and surgeries involving the femoral neck propose a significant risk of injury to the LCFA with the crippling consequence of femoral head avascular necrosis, which affects 10,000 to 20,000 patients in the United States per year [14].

In the context of analgesia following knee and hip replacement surgeries, femoral nerve blocks administered without knowledge of anatomical LCFA variations could result in damage to the artery, as the femoral nerve is located anteriorly [15]. According to the 2021 American Joint Replacement Registry Report, over 2.24 million knee and hip arthroplasties were completed from 2012-2020; however, this number only represents 40% of such procedures in the United States during this period, further illustrating the prevalence of surgeries occurring daily [16]. Moreover, the LCFA is an astounding and clinically significant structure. Discovery and documentation of its variable anatomical course improve clinical practice and patient outcomes for various surgical procedures, including CABG, reconstructive surgeries, procedures involving the hip and knee joints [2], and surgeries of the anterior thigh [15], among others. Curiosity regarding the variability of anatomical structures is imperative for physicians in modern practice, as exemplified by the LCFA.

## Conclusions

The typical course of the lateral circumflex femoral artery (LCFA) involves its branching from the deep femoral artery (DFA), supplying vital soft tissues encompassing the hip and knee joints, tensor fasciae latae and quadriceps femoris muscles, and the skin of the anterolateral thigh. However, an unusual discovery was made in our cadaveric study: a secondary LCFA originating superiorly from the common femoral artery (CFA) that also maintained its ascending, lateral, and descending branches. Despite documented variations, this duplicated LCFA and its branches present a unique and, as far as we know, unprecedented observation. While this anatomical variance may not signify an inherent pathology, it remains crucial for surgeons to acknowledge this anomaly during surgical procedures to mitigate potential complications and iatrogenic injuries.
